# Astrocyte-based cell therapy: new hope for amyotrophic lateral sclerosis patients?

**DOI:** 10.1186/s13287-018-1006-y

**Published:** 2018-09-26

**Authors:** Luis Barbeito

**Affiliations:** Institut Pasteur de Montevideo, Montevideo, Uruguay

## Abstract

Amyotrophic lateral sclerosis (ALS) is a fatal paralytic disease with no cure or treatment to stop disease progression. Because ALS represents an urgent unmet medical need, a significant number of therapeutics are being tested in preclinical and clinical studies. A recent publication in *Stem Cell Research & Therapy* by Izrael and colleagues reports about embryonic stem cell-derived astrocytes as a potential cell therapy for ALS. Such cells behave as highly trophic “young astrocytes”, being able to delay disease onset and prolong survival when injected intrathechally in murine models of ALS overexpressing the SOD1^G93A^ mutation. The safety and therapeutic potential of these cells are currently being evaluated in a clinical trial in ALS patients. This commentary discusses the mechanisms of action and potential therapeutic effects of these “young astrocytes” in ALS.

Extensive evidence supports the notion that the pathogenesis of neurodegenerative disease involves a disruption of the normal functional interplay between vulnerable neurons and non-neuronal cells, with a key neurotoxic role played by phenotypically abnormal astrocytes and microglia as well infiltrating immune cells [1, 2].

In particular, astrocytes become toxic for motor neurons in amyotrophic lateral sclerosis (ALS), losing their ability to provide trophic, metabolic, and synaptic support to cultured motor neurons (reviewed in [3]). This toxicity has been described for rodent-derived ALS astrocytes subjected to oxidative stress or expressing ALS-linked SOD1 mutations, as well as neural stem cell-derived astrocytes from ALS patient [4, 5]. Thus, astrocyte dysfunction appears to be a well-recognized pathologic phenomenon in ALS.

Several mechanisms have been proposed to explain the deleterious activity of astrocytes in ALS: release of inflammatory and pro-apoptotic mediators [6], induction of oxidative and nitrative stress, as well as glutamate- and ATP-mediated excitotoxicity [7]. In this context, astrocytes represent potential therapeutic targets in ALS through approaches involving the reversal of deleterious activities or restoration of trophic support to motor neurons by means of cell therapy.

Izrael and collaborators [8] developed an original and promising cell therapy approach to ALS using human embryonic stem cell (hESC)-derived astrocytes (hES-AS) [8]. They show that, after intrathecal transplantation of “young” astrocytes into a murine NSG model, the cells distribute throughout the neural axis and survive for a long time attached to Pia mater, in close proximity to central nervous system parenchyma without penetrating the cellular microenvironment of the grey or white matter. The transplanted cells are safe, retaining their astrocytic phenotype and functions without unwanted transformation or uncontrolled proliferation. These findings suggest that delivery of young and healthy astrocytes could compensate for the neurotoxic function of endogenous astrocytes in ALS. According to functional and secretome analysis, the young astrocytes secrete soluble factors that promote the growth of axons and neuron survival. In addition, the astrocytes were found to uptake extracellular glutamate and protect motor neurons from oxidative stress damage, thus providing a plausible mechanism of action based on the enhancement of astrocyte activity that is lost during ALS. The results are in accordance with a previous report showing transplantation of astrocyte precursors delayed progression of mutant SOD1-mediated disease in rodents [9] .

One remarkable feature of this study is the development of a method to produce large quantities of clinical grade astrocyte progenitor cells that can be expanded and stored frozen in a controlled and reproducible manner. These cells can be induced to final differentiation before transplantation, thus becoming a valid cell therapy approach ready to be tested in clinical trials.

Izrael and colleagues study raises several questions and speculations about the significance of therapy using young astrocytes in ALS patients. Firstly, while multiple pathogenic pathways involving neural and non-neural cells are thought to mediate neurodegeneration in ALS, it is intriguing how transplanted young astrocytes protect the host. Is their protective activity solely mediated by the production and release of soluble factors that permeate the degenerated microenvironment to prevent disease? The authors propose a central trophic mechanism of action underlying protection by young and committed astrocytes in murine models of ALS. The young astrocytes were found to secrete factors with supporting activity on neurons as well as several antiproteases which could remodel extracellular matrix, thereby shedding new light on the possible mechanisms of action underlying the observed therapeutic effect in ALS models. These factors include Osteopontin (OPN/SSP1), which was found to stimulate regeneration of motor axons, TIMP-1 and 2 inhibitors of MMP9 and other matrix metalloproteases which play a major role in preventing degradation of extracellular matrix (ECM), CXCl-16 chemokine, which was found to increase neuron survival, Clusterin, Midkine, and the well-studied GDNF, BDNF, and VEGF trophic factors. However, other complementary mechanisms could also be involved, including the production of exosomes or microvesicles delivering tiny amounts of other mediators or microRNAs, which may have been under the detection limit in the current secretome analysis. It is also possible that young implanted astrocytes could antagonize oxidative stress in ALS-affected regions by releasing glutathione as well as other antioxidants into the extracellular medium. Thus, young transplanted astrocytes would likely behave as protective A2 astrocytes previously characterized in rodents [10]. By extension, one could anticipate additional protective effects stimulating re-myelination and new synapse formation.

Age-dependent senescent astrocytic phenotypes have been shown to lose their trophic support of motor neurons and promote inflammation [11]. Because astrocyte senescence can be reversed by GDNF, it can be anticipated that transplanted young astrocytes may also influence deleterious activity of abnormal glial cells in the ALS cellular microenvironment.

Another important question about young astrocytes is whether these cells represent a potential cellular therapy to target neuroinflammation in ALS. Evidence indicates that inflammatory or immune mechanisms also influence the degeneration of the cellular microenvironment, with discrete infiltration of T cells, monocytes, and mast cells [12]. Potentially, young astrocytes may modulate specific pathways of innate or adaptive immune responses that accelerate disease progression. They also secrete a significant number of metalloprotease inhibitors that could mediate an anti-inflammatory effect.

## Conclusions

The two current FDA-approved drugs for ALS, riluzole and edaravone, only modestly attenuate disease progression. Both these small molecule drugs are based on a single mechanism of action. ALS is a multifactorial disease and therapeutic approaches should take into account the multiplicity of mechanisms that underlie motor neuron degeneration in this disease. Thus, young astrocytes that act through multiple mechanisms of action to treat the broad pathological aspects of the disease are more likely to be effective. Would cell replacement with young astrocytes prevent or revert neuronal damage or the emergence of activated astrocytes in neurodegenerative or demyelinating diseases other than ALS? Future studies should test these possibilities.

## References

[1] Barbeito LH (2004). A role for astrocytes in motor neuron loss in amyotrophic lateral sclerosis. Brain Res Brain Res Rev.

[2] McGeer PL, McGeer EG (2002). Inflammatory processes in amyotrophic lateral sclerosis. Muscle Nerve.

[3] Izrael M (2018). Astrocytes in pathogenesis of neurodegenerative diseases and potential translation into clinic. Astrocyte - Physiology and Pathology.

[4] Nagai M (2007). Astrocytes expressing ALS-linked mutated SOD1 release factors selectively toxic to motor neurons. Nat Neurosci.

[5] Amanda M Haidet-Phillips, Mark E Hester, Carlos J Miranda, Kathrin Meyer, Lyndsey Braun, Ashley Frakes, SungWon Song, Shibi Likhite, Matthew J Murtha, Kevin D Foust, Meghan Rao, Amy Eagle, Anja Kammesheidt, Ashley Christensen, Jerry R Mendell, Arthur H M Burghes, Brian K Kaspar, (2011) Astrocytes from familial and sporadic ALS patients are toxic to motor neurons. Nature Biotechnology 29(9):824–828. 10.1038/nbt.1957 PMC3170425 21832997

[6] Vargas MR (2006). Increased glutathione biosynthesis by Nrf2 activation in astrocytes prevents p75NTR-dependent motor neuron apoptosis. J Neurochem.

[7] Gandelman M (2010). Extracellular ATP and the P2X7 receptor in astrocyte-mediated motor neuron death: implications for amyotrophic lateral sclerosis. J Neuroinflammation.

[8] Izrael M (2018). Safety and efficacy of human embryonic stem cell-derived astrocytes following intrathecal transplantation in SOD1(G93A) and NSG animal models. Stem Cell Res Ther.

[9] Lepore AC (2008). Focal transplantation-based astrocyte replacement is neuroprotective in a model of motor neuron disease. Nat Neurosci.

[10] Clarke LE (2018). Normal aging induces A1-like astrocyte reactivity. Proc Natl Acad Sci U S A.

[11] Thomsen Gretchen M., Avalos Pablo, Ma Annie A., Alkaslasi Mor, Cho Noell, Wyss Livia, Vit Jean-Philippe, Godoy Marlesa, Suezaki Patrick, Shelest Oksana, Bankiewicz Krystof S., Svendsen Clive N. (2018). Transplantation of Neural Progenitor Cells Expressing Glial Cell Line-Derived Neurotrophic Factor into the Motor Cortex as a Strategy to Treat Amyotrophic Lateral Sclerosis. STEM CELLS.

[12] Graves M, Fiala M, Dinglasan Lu Anne, Liu N, Sayre J, Chiappelli F, van Kooten C, Vinters H (2009). Inflammation in amyotrophic lateral sclerosis spinal cord and brain is mediated by activated macrophages, mast cells and T cells. Amyotrophic Lateral Sclerosis and Other Motor Neuron Disorders.

